# Granulomatosis With Polyangiitis in a Pediatric Male

**DOI:** 10.7759/cureus.12055

**Published:** 2020-12-13

**Authors:** Florentino Saenz Rios, Sandhya Devaraj, Giri Movva, Hari Movva, Quan D Nguyen

**Affiliations:** 1 Radiology, University of Texas Medical Branch, Galveston, USA; 2 Family Medicine, The University of Texas Health Science Center, Tyler, USA; 3 Medicine, University of Texas Rio Grande Valley School of Medicine, Edinburg, USA

**Keywords:** pediatrics, polyangiitis, wegener, vasculitides

## Abstract

Significant eosinophilia is a prominent feature in Churg-Strauss syndrome but has not been described in granulomatosis with polyangiitis (GPA) in a pediatric patient. We present a biopsy case that confirmed granulomatosis with polyangiitis with significant eosinophilia > 30% on the initial presentation. Etiologies that could account for eosinophilia were excluded during workup. The patient's presentation of pulmonary alveolar hemorrhage, conjunctivitis arthritis, high-titer cytoplasmic antineutrophil, PR3-ANCA antibodies, and cytoplasmic antibodies (cANCA) was consistent with a clinical picture of atypical GPA in a pediatric patient. This case presents a rare opportunity not only due to GPA’s low incidence in the pediatric population but due to the unusual nature of significant eosinophilia in GPA.

## Introduction

Granulomatosis with polyangiitis (GPA) is a multisystem necrotizing granulomatous vasculitis of the small to medium-sized vessels that usually presents between the ages of 40 and 60 years [1-3]. GPA’s classic presenting triad of nasal, pulmonary, and renal involvement holds in the pediatric population, with milder presentations fever, malaise, and weight loss being more prevalent [1-4]. Wegener’s eosinophilic variant was first described in 1954 by Godman and Churg, with only a limited number of cases since then describing this phenomenon [5]. Notably, a pediatric case of GPA is a rare opportunity presenting with a pediatric occurrence rate of 1:1,000,000; however, such a presentation, in addition to the presence of significant eosinophilia, has yet to be described in the literature [3]. This case report presents a rare opportunity wherein a pediatric case of variant GPA with eosinophilia was diagnosed amid the COVID-19 pandemic.

## Case presentation

 A 17-year-old African American male presented to the emergency department with arthralgias, fever, nausea, and vomiting that was worsening for the last four days. He had been seen at a rural ED twice in the last two weeks for headaches and arthralgias, where he was diagnosed with viral upper respiratory tract infection and tested COVID-19 negative via polymerase chain reaction (PCR). ED workup showed a white blood cell count of 21,000, d-dimer of 1500, and a sedimentation rate of 82 shown in Table 1.

**Table 1:** Additional Diagnostic Studies and Their Results for ED Admission SARS-CoV-2 PCR: severe acute respiratory syndrome coronavirus 2 polymerase chain reaction

Study	Study Results	Normal Range	Hospital Day Ordered
SARS-CoV-2 PCR	Negative	Negative	Prior to Admission
WBC Count	21,000	3.8-10.6k	ED Admission
D-Dimer	1502	<500ng/mL	ED Admission
Sedimentation Rate	82	0-15 males 0-20 females	ED Admission
Urinalysis	• 1000+ protein • small leukocyte esterase • large blood (10-20 RBCs)	• No protein • No leukocyte esterase • No blood	ED Admission
CT Abdomen/Pelvis	Bilateral patchy infiltrates in the lower lungs.		ED Admission

Urine analysis was remarkable for 1000+ protein, small leukocyte esterase, and large blood, with 10-20 red blood cells. Abdominal/pelvic CT showed no remarkable findings.

Repeat COVID-19 PCR confirmed the patient’s negative status. This presentation led to the patient being admitted for a tentative diagnosis of pneumonia and urinary tract infection.

**Figure 1:**
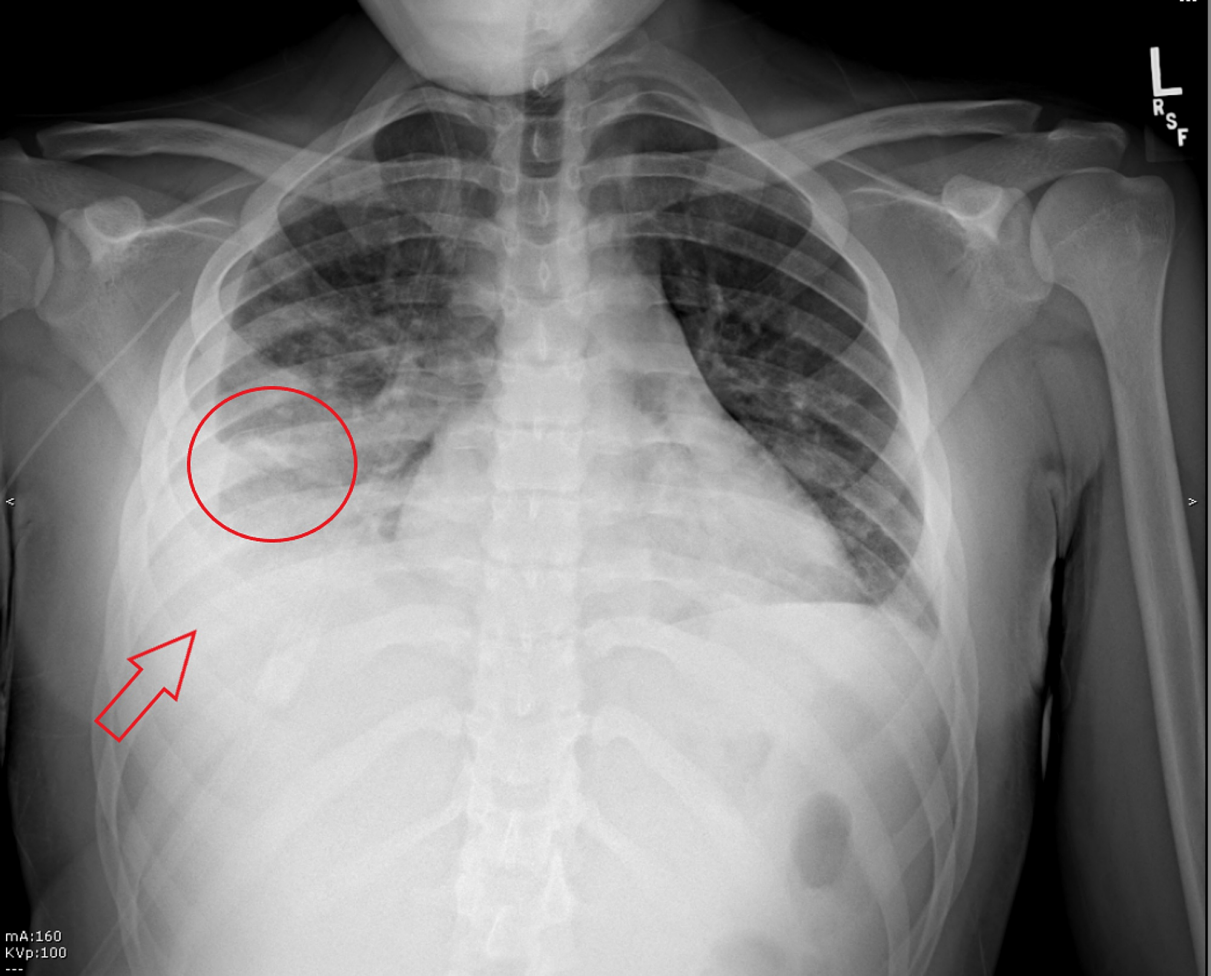
Frontal radiograph demonstrates patchy opacification (circle) with a relative central lucency (circle). Additionally, there is a small-moderate volume right pleural effusion (arrow) with adjacent compressive atelectasis.

On hospital day 1, notable physical examination findings included bilateral scleral injection, clear oropharynx with no mucosal changes, clear lungs bilaterally, and mild epigastric tenderness. Overnight d-dimer rose to 2880 (Table 2), prompting a repeat chest x-ray in the morning with a CT angiography to assess for the development of an infectious process in addition to pulmonary embolism (PE). A repeat x-ray showed the presence of cavitary lesions with an overlying effusion of the right lower lobe (Figure 1).

**Table 2:** Additional Diagnostic Studies and Their Results for Day 1 SARS-CoV-2 PCR: severe acute respiratory syndrome coronavirus 2 polymerase chain reaction

Study	Study Results	Normal Range	Hospital Day Ordered
SARS-CoV-2 PCR	Negative	Negative	Hospital Day 1
Urine Legionella Antigen	Negative	Negative	Hospital Day 1
Urine Gonorrhea/Chlamydia Screen	Negative	Negative	Hospital Day 1
Urine Drug Screen	Negative	Negative	Hospital Day 1
D-Dimer	2880	<500ng/mL	Hospital Night 1

**Figure 2:**
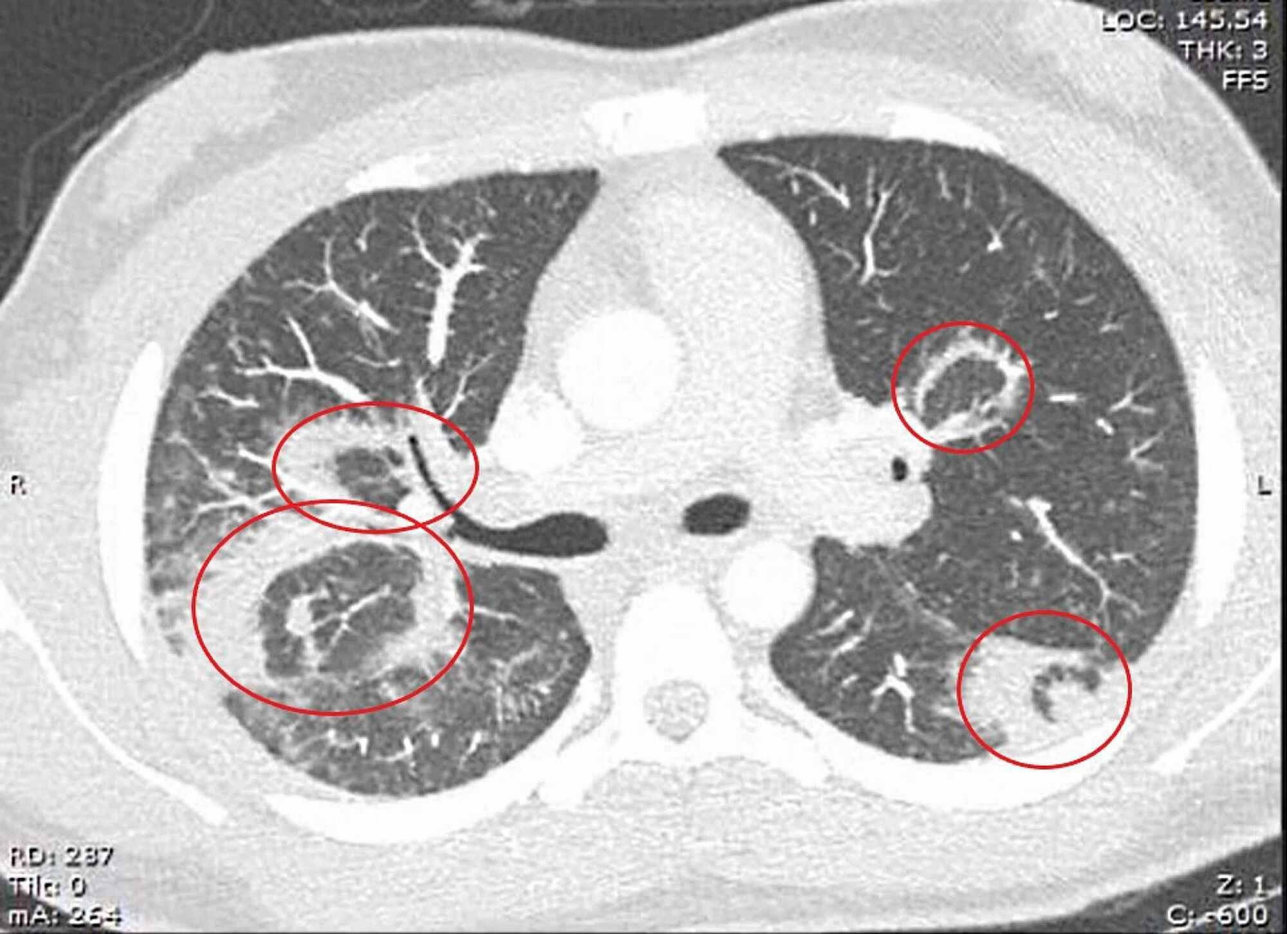
CT chest axial view demonstrates scattered patch opacities (circles).

**Figure 3:**
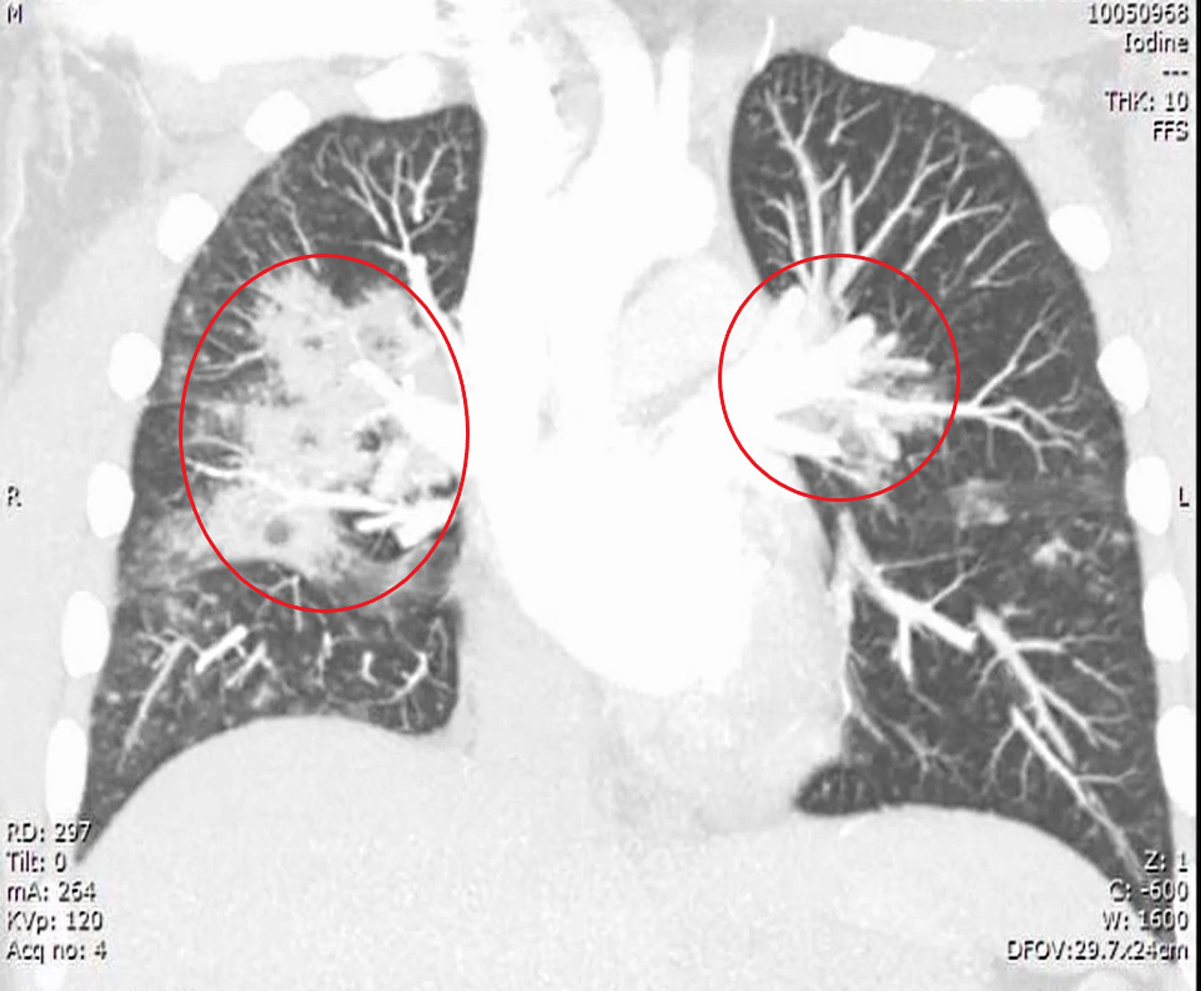
CT chest single slice coronal view maximum intensity projection image demonstrates bilateral perihilar focal consolidation, left (circle) greater than right (circle).

Repeat CT imaging now showed the presence of infiltrates and cavitary lesions in both lungs with no evidence of underlying PE (Figures 2, 3).

The infectious disease team (ID) noted a 31% eosinophilia, prompting several parasitic serologies, an HIV screen, and rheumatological studies including IL-6, CD-4, c-ANCA, and ANA. The patient was started on low dose solumedrol pending lab results. On hospital day 2, the patient improvement was noted alleviation of arthralgias in addition to his WBC count decreasing to 14k with only 5% eosinophilia. Supportive care with antipyretics and solumedrol continued.

On hospital day 3, the patient desaturated to 83% on room air and was started on 1 liter (L) nasal cannula (NC). Creatinine remained at 1.6 from 1.8 on admission, despite 48 hours of IV hydration. Kidney biopsy was delayed due to an elevated d-dimer of 3500 from hospital day 2 (Table 3). INR check resulted in 2.0, prompting rescheduling to the following day. Midday d-dimer was noted to be 7000 (Table 3), prompting a rheumatological consult. Bronchoscopy was scheduled along with kidney biopsy the following day. On hospital day 4, the patient, now on 5L NC, was taken to bronchoscopy, where significant alveolar hemorrhage was noted. The profuse bleeding prompted a bronchoalveolar lavage; the biopsy was not completed. The patient was unable to maintain adequate oxygenation and was ventilated and transferred to a tertiary care pediatrics center by helicopter. A kidney biopsy performed at the tertiary care facility confirmed the diagnosis with PR3-ANCA associated glomerulonephritis in a necrotizing and crescentic pattern being noted.

**Table 3:** Additional Diagnostic Studies and Their Results for Days 2 and 3 SARS-CoV-2 PCR: severe acute respiratory syndrome coronavirus 2 polymerase chain reaction

Study	Study Results	Normal Range	Hospital Day Ordered
SARS-CoV-2 PCR	Negative	Negative	Hospital Day 2
WBC Count	14,000 w/5% eosinophils	4500-11,000/mm^3^	Hospital Day 2
D-Dimer	3500	<500ng/mL	Hospital Day 2
INR	2	≤1.1	Hospital Day 3
D-Dimer	7000	<500ng/mL	Hospital Day 3

## Discussion

GPA in a pediatric patient is a rare but not unheard of occurrence; however, such a case with an atypical presentation of eosinophilia in a pediatric patient has not yet been recorded within the literature. GPA may occur at any age; however, over 85% of cases occur past the age of 19 [2] with the peak age of incidence being ages 40-60 years of age [1-3]. In adults, GPA has a reported incidence rate of approximately 1:100,000, which stands in direct contrast to the pediatric population where the reported incidence of GPA is approximately 1:1,000,000 [3]. Commonly, Churg-Strauss vasculitis, or eosinophilic granulomatosis with polyangiitis (eGPA), is marked by significant eosinophilia. The presence of eosinophilia in GPA is atypical, with underlying etiologies needing to be ruled out before attributing the eosinophilia to the GPA.

Notable features seen in our patient that coincide with other atypical GPA cases are the presence of significant pulmonary alveolar hemorrhage, conjunctivitis arthritis, high-titer cytoplasmic antineutrophil cytoplasmic antibodies (cANCA), and proteinase-3 antibodies. The eosinophilic variant of GPA is characterized by significant tissue eosinophilia in the absence of asthma, atopy, or pANCA antibodies [6]. The patient’s history was negative for recent travel, asthma, atopy, or underlying pneumonia on the visit. The patient’s titers also did not reveal the presence of an underlying pANCA associated Churg-Strauss vasculitis. The patient’s preliminary CT was consistent with GPA, showing the presence of consolidations (Figures 2, 3) and scattered patch opacities (Figure 2) in the absence of positive sputum cultures. Such a presentation of GPA may be an early sign of polyangiitis overlap syndrome (POS) between GPA and eGPA, a phenomenon first described by Leavitt and Fauci in 1986 [7]. While Leavitt and Fauci set no set criteria for POS, the patient’s lack of asthma and atopy, classic signs of eGPA, create a clinical picture favoring an atypical presentation of GPA over POS [7].

Peripheral blood eosinophilia has only been described as an uncommon finding in GPA with a possible association with pulmonary hemorrhage in patients [6]. The limited amount of cases described in the literature make assessing if eosinophilia is associated with more severe presentations difficult. However, the overall diagnostic workup and treatment for GPA remain the same. It is worth noting that when treating pediatric patients, one must consider that the cANCA titer level is not indicative of the disease burden, as seen in our patient who developed sudden alveolar hemorrhage requiring ventilation. This illustrates a rare and extreme complication of GPA in the pediatric population [8]. Management of GPA consists of a combination of corticosteroids, cyclophosphamide, and rituximab to suppress the patient’s immune system, inducing a state of remission [3]. Once remission is induced, the patient is followed up with a maintenance dose of steroids and other disease-modifying agents. Early diagnosis of GPA is necessary to ensure patient survival due to GPA’s one-year mortality approaching 90% if left untreated [3]. In contrast, with proper maintenance therapy, a patient’s five-year survivability will approach 90% [3]. Once remission of GPA is reached, the main concerns would be the chance of relapse and therapy-related complications due to the chronic corticosteroids and immunosuppressants [2,3].

## Conclusions

This pediatric patient had an atypical presentation for GPA; both the presence of notable eosinophilia and eventual alveolar hemorrhage are uncommon. The incidence of GPA is exceeding rare in the pediatric population, at 1:1,000,000. The wide array of chief complaints over his four emergency department presentations delayed the diagnosis, as did the overlapping symptoms with the novel SARS-CoV. 

## References

[1] Martinez F, Chung JH, Digumarthy SR (2011). Common and uncommon manifestations of Wegener granulomatosis at chest CT: radiologic-pathologic correlation. Radiographics.

[2] Ayala de la Cruz MdC, González Díaz R, López Lara ND ( 2003). Wegener's granulomatosis. Report of a pediatric case and review of the literature. [Article in Spanish]. Rev Alerg Mex.

[3] Bohm M, Gonzalez Fernandez MI, Ozen S (2014). Clinical features of childhood granulomatosis with polyangiitis (wegener’s granulomatosis). Pediatr Rheumatol Online J.

[4] Arulkumaran N, Jawad S, Smith SW, Harper L, Brogan P, Pusey CD, Salama AD (2011). Long- term outcome of paediatric patients with ANCA vasculitis. Pediatr Rheumatol Online J.

[5] Godman GC, Churg J (1954). Wegener's granulomatosis: pathology and review of the literature. AMA Arch Pathol.

[6] Potter MB, Fincher RK, Finger DR (1999). Eosinophilia in Wegener's granulomatosis. Chest.

[7] Leavitt RY, Fauci AS (1986). Polyangiitis overlap syndrome: classification and prospective clinical experience. Am J Med.

[8] Filocamo G, Torreggiani S, Agostoni C, Esposito S (2017). Lung involvement in childhood onset granulomatosis with polyangiitis. Pediatr Rheumatol.

